# Determinants of cervical cancer screening utilization among women attending health facilities of Dessie town, Northeast Ethiopia

**DOI:** 10.1186/s12885-022-10447-0

**Published:** 2022-12-20

**Authors:** Tilahun Assefa, Mastewal Arefaynie, Wondwosen Mebratu, Anissa Mohammed, Elsabeth Addisu, Natnael Kebede

**Affiliations:** 1 Department of Epidemiology and Biostatistics, School of Public Health, College of Medicine Health Sciences, Wollo University, Dessie, Ethiopia; 2 Department of Reproductive Health, School of Public Health College of Medicine Health Sciences, Wollo University, Dessie, Ethiopia; 3 Department of Health Promotion, School of Public Health College of Medicine Health Sciences, Wollo University, Dessie, Ethiopia

**Keywords:** Cervical cancer, Determinants, Screening

## Abstract

**Introduction:**

Despite the higher burden of cervical cases, screening programs in highly affected developing countries remained low. This made the disease to be present at an advanced stage which is almost always fatal, causing enormous pain and suffering for the individual and having significant adverse effects on the welfare of their families and community. Thus, this study aimed to assess determinants of cervical cancer screening utilization among women attending health facilities in Dessie Town, Northeast Ethiopia.

**Methods:**

An institution-based unmatched case–control study design was employed on 430 women (146 cases and 284 controls) at selected health facilities of Dessie town, South Wollo Zone, from July 1/2020 to August 30/2020. Cases were selected for all women screened for cervical cancer during the data collection period until the required sample size was attained and using a consecutive sampling technique, every 3 participants from women who come for services other than cervical cancer screening. were included as controls. Pretested and structured questionnaires were used to collect the data. Data were analyzed by SPSS version 25 software. Bivariable and multivariable logistics regression analysis was done. An adjusted odds ratio with 95% CI was estimated to measure the strength of the association. The level of statistical significance was declared at a *p*-value < 0.05.

**Result:**

Age group of 35 and more [AOR = 11.52(6.09–21.77)], being a private employee [AOR = 4.67(2.41–9.03)], having symptoms of vaginal bleeding or pelvic pain or postcoital bleeding or vaginal discharge [AOR = 3.08(1.37–6.95)], being recommended by a physician for screening [[AOR = 3.07(1.45–6.49)] and positive attitude towards cervical cancer screening [AOR = 5.3(2.8–10.59)] were determinants of cervical cancer screening.

**Conclusion:**

Age group of 35 and more, current occupation as a private employee, having symptoms of cervical cancer, being recommended by a physician for screening, and positive attitude towards cervical cancer screening were determinants of cervical cancer screening utilization.

**Supplementary Information:**

The online version contains supplementary material available at 10.1186/s12885-022-10447-0.

## Introduction

Cervical cancer screening is the systematic application of a test to identify cervical abnormalities in an asymptomatic population. It is testing of women at risk of cervical cancer that aims to detect precancerous changes, which, if not treated, may lead to cancer. Women targeted for screening may feel perfectly healthy and see no reason to visit health facilities [1]. Women who have abnormalities on screening need follow-up, diagnosis, and possibly treatment, to prevent the development of cancer or to treat cancer at an early stage [2].

Screening service is recommended to be performed for sexually active females with age 21–65 years. Screening after a woman's age greater than 65 years old is not necessary [3]. From these screening tests, Pap smear and visual inspection with acetic acid are the commonest [4].

A Pap test is performed by scraping off some cells from the surface of the cervix using a spatula and a brush and then cancer cells are identified through a microscopic examination. It identifies women who may have pre-invasive or early cancerous changes. It is recommended that a Pap test for cervical screening would be conducted within three years of the initiation of sexual activity or at the age of 21 whichever occurs earlier. Rescreening is reduced to every two to three years until the age of 69 if the first two smears are normal at the discretion of the physician [4].

In areas where Pap smear screening is not available or affordable, visual inspection of the cervix, using acetic acid to highlight precancerous lesions so they can be viewed with the naked eye, is also currently available [5].

Cervical cancer is the easiest gynecologic cancer to prevent with effective screening practices. The availability of relatively simple screening tests to detect premalignant lesions and the availability of the human papillomavirus vaccine made the proverb 'prevention is better than cure' appropriate for cervical cancer [6]. Additionally, cervical cancer has a long premalignant period that provides the opportunity to screen and treat it before it becomes invasive cervical cancer [7]. Along with this, it is largely preventable by effective screening programs. Effective screening can reduce the risk of developing invasive cervical cancer by 90% [3].

The large decline in cervical cancer mortality in developed countries had been attributed to widespread screening. It is 80% in Austria and Luxemburg, 79% in Georgia, and 73% in Brazil [2].

Despite the higher burden of the cervical case and the fact that easily preventable, the coverage of the screening program for highly affected women in developing countries remained at less than 5%. In Nigeria and Kenya, the rate of screening program coverage was below 5.3% and 3.2% respectively. In West Africa, Less than 1% of women in four countries had ever been screened [2].

In Ethiopia, studies have shown that uptake of cervical cancer screening is very low, for example, in the towns of Arbaminch (5.9%) [8], Addis Abeba (3.5%) [9], Mekelle (10.7%) [10] and Dessie (11%) [11]compared to the National cervical cancer prevention strategic plan (80%) set by Federal Ministry of Health of Ethiopia [12].

Low cervical cancer screening coverage in developing countries made the disease to be present at an advanced stage that requires extensive treatment modalities like surgery, radiotherapy, and chemotherapy and has a markedly diminished chance of success In addition, these treatment modalities may be lacking altogether, or too expensive and inaccessible for many women and if left untreated, invasive cervical cancer is almost always fatal, causing enormous pain and suffering for the individual and having significant adverse effects on the welfare of their families and community [1]. The 2009 World Health Organization report depicts the urgent need for action to increase the utilization of cervical cancer screening very clearly. The report suggests that in Ethiopia, the rate of age-adjusted cervical cancer is around 27.5 per 100,000 patients. The increment in new cases per year is 7619 and deaths per year are reported as 6081. Without urgent attention, deaths due to cervical cancer are projected to rise by almost 25% over the next 10 years [13]. The World Health Organization (WHO) and the American Cancer Society (ACS) recommended that all age-eligible women should have cervical cancer screening at least once every five years [14].

Knowledge and attitude towards cervical cancer screening, perception of susceptibility, age, occupation, income, pregnancy history, and ever-use modern contraceptives are key determinants of self-reported uptake of cervical screening revealed by previous studies in other regions [15–17]. Very little is known about cervical cancer screening and physician recommendations. So this thesis is regarding the determinants of cervical cancer screening utilization.

Identifying the determinants of cervical cancer screening utilization could result in one of the most significant successes to decrease women's mortality and morbidity related to cervical cancer in Dessie town as well as in the country by achieving the national cervical cancer prevention strategic plans set by the Federal Ministry of Health. Additionally, it can be used as a tool in achieving a health sector transformation plan and national sexual and reproductive health strategy.

## Methods and materials

### Study setting, design, and period

An institution-based unmatched case–control study was employed among 442 women of age 15 and above at the health Centers of Dessie town, from March to April/ 2020. It is better to use unmatched case control because it enhances the power of the study. Seventeen health institutions are available in Dessie town providing cervical cancer screening services to the community.

### Population and sample

All women of age 15 and above who have been screened for cervical cancer at health facilities of Dessie town and all women of age15 and above who have been presenting at the health facilities of Dessie town for any service other than cervical cancer screening were the target population for case and control group respectively. All women of age15 and above, who presented at the selected health facilities of Dessie town for cervical cancer screening during the data collection period, and All women of age 15 and above, who presented at the selected health facilities of Dessie town for any service other than cervical cancer screening during data collection period who fulfilled the inclusion criteria were considered as the study population for case and control group respectively. For Cases: Women who are ever screened for cervical cancer previously and Controls: women who are in labor and women who are critically ill were excluded.

### Sample size determination

The sample size was determined using Epi info version 7 (StatCalc—Sample Size and Power—Unmatched Case–Control Study); by taking the assumptions of 95% confidence level, 80% power, the ratio of controls to cases 2, percent of controls exposed from previous studies and 10% non-response rate. The largest sample size for the objective calculated using Epi info considering 10% non-response was 430. Since the ratio of controls to cases were 2, the final sample size was 146 for cases and 284 for controls.

### Sampling procedure

Of all institution that provides cervical cancer screening, 6 were selected using a lottery method. The selected health institutions were: Selam hospital, Ethio hospital, Family Guidance Association of Ethiopia, Dessie branch, Mary Stops international clinic, Segno Gebeya health center, and Dr. Misganaw clinic. The total sample size was allocated using proportional allocation to size according to the proportion of average monthly client flow for cervical cancer screening reviewed from the registration book and the number of respondents required for the study in each facility was determined. Cases were selected for all women screened for cervical cancer during the data collection period until the required sample size was attained and every 3 participants, who have been presenting at the health facilities of Dessie town for any service other than cervical cancer screening, were included as controls. After every case, two controls were selected using consecutive sampling.

### Operational definitions

#### Cervical cancer screening

A procedure that is performed to identify the presence of abnormal cells in the cervix. So a woman screened for cervical cancer at the time of data collection is categorized as cervical cancer screening utilized and if not cervical cancer screening not utilized.

#### Knowledge assessment

The respondents were asked 24 questions regarding knowledge of cervical cancer screening. One question had one point. summed up to get the score and used as a continuous variable for further analysis.

#### Attitude assessment

Ten questions measuring attitude were asked of the respondents. Those who had a score less than the mean were considered to have a negative attitude. summed up to get the score and used as a continuous variable for further analysis.

### Data collection procedure

The data was collected by using a structured questionnaire developed through a review of documents, guidelines, and manuals related to unintended pregnancy and most of the questions were close-ended. 6 trained health professionals carried out data collection, 2 BSC nurses, 2 midwives, 2 health officers from the health centers, and one supervisor after one day of training on the data collection and interview techniques by the principal investigator three days after the actual study was done.

### Data quality control

Initially, a data collection tool was prepared and the questionnaire was translated into the Amharic Language which is used as the national language. Before the actual data collection, the questionnaire was submitted to the subject experts to check the content validity. Then, the questionnaires had been adopted according to the suggestions and comments of the subject's expertise and proceed to the Pre-test in the FGAE Kombolcha branch, which was one of the public health facilities in the study area, by taking 5% (7 cases and 14 controls) of the sample size one week before data collection period. Additional modifications and adjustments in the sequence of knowledge questions and wording of the questionnaire were made based on the results of the pre-test. During data collection, data collectors were supervised daily by the supervisors to monitor the data collection process and check for the completeness and logical consistencies of the data and reported to the principal investigator on a daily base appropriate feedback had been given.

### Data processing and analysis

After the Data was collected, it was coded, entered, cleaned using Epidata v4.4, and analyzed using SPSS version 25, and Data was summarized by using tables and figures. All variables with a *p*-value ≤ 0.25 during bi-variable analysis were considered for multivariable logistic regression analysis to control for all possible confounders and to identify factors associated with the outcome variable. The strength of statistical association was measured by adjusted odds ratio and 95% confidence intervals and a *p*-value < 0.05 was considered statistically significant. The goodness of fit of the final model was checked using the Hosmer and Lemeshow test of best fit with a large *p*-value.

## Result

### Socio-demographic characteristics of participants

Out of 442 study participants, 430 (146cases and 284 controls) participated in the study making a response rate of 97%and there was no missing data. One-third of participants, 22(151%) cases, and 207 (72.9%) controls were in the age group of < 35 years. The majority of the participants, 140(95.8%) cases and 245(86.2%) controls were married. 64 (43.8%) cases and 127 (44.7%) controls attended secondary school.

Regarding residence, 101 (69.2%) cases and 139 (48.9%) controls were residing in urban. The highest number of respondents, 47 (32.2%) cases, and 201 (70.77%) controls were housewives. Regarding average monthly income, 109 (74.6%) cases and 116 (40.8%) controls earn more than 2000 birr (Table 1).

### Behavioral, gynecologic, and obstetric history of participants

Among the study subjects majority of them 145(99.3%) of the cases and 280(98.5%) of the controls, their age at first sex was greater than 17 years old, 142(97.3%) of the cases and 243 (85.6%) of the controls had ever given birth. 131(89.7%) of the cases and 216(76%) of the controls had ever used contraceptives, 140(95.9%) of the cases and 266(93.6%) of the controls had ever tested for HIV. 99 (67.8%) of the cases and 152(53.5%) of the controls had greater than or equal two sexual partners(Table 2).

**Table 1:** Socio-demographic characteristics of women attending health facilities of Dessie town, Northeast Ethiopia 2020

Variable	Characteristics	Cervical cancer screening		Total
		**Screened (cases)**	**Not screened (controls)**	
Age in years	< 35	22(15.1)	207(72.9)	229(53.2)
	> = 35	124(84.9)	77(27.1)	201(46.8)
Religion	Orthodox	66(45.2%)	128(45.1%)	194(45.1%)
	Muslim	71(48.6%)	134(47.2%)	205(47.7%)
	Protestant	9(6.2%)	22(7.7%)	31(7.2%)
Residence	Urban	101(69.2%)	139(48.9%)	240(55.8%)
	Rural	45(30.8%)	145(51.1%)	190(44.2%)
Educational status	No formal education	16(11%)	52(18.3%)	68(15.8)
	Primary	31(21.2%)	74(26.1%)	105(24.4%)
	Secondary	64(43.8%)	127(44.7%)	191(44.4%)
	College and above	35(24%)	31(10.9%)	66(15.4%)
Occupation	Housewife	47(32.2%)	201(70.8%)	248(57.7%)
	Gov.t employee	20(13.7%)	23(8.2%)	43(10%)
	Private employee	79(54.1%)	60(21%)	139(32.3%)
Average monthly income	< 1000	8(5.5%)	95(33.4%)	103(23.9%)
	1000–1999	29(19.8%)	73(27.7%)	102(23.8%)
	≥ 2000	109(74.7%)	116(40.9%)	225(52.3%)

**Table 2:** Behavioral, gynecologic, and obstetric history of women attending health facilities of Dessie town, Northeast Ethiopia 2020

Variable	Characteristics	Cervical cancer screening		Total
		**Screened(cases)**	**Not screened (controls)**	
Have you ever tested for HIV	Yes	140(95.9%)	266(93.6%)	406(94.4%)
	No	6(4.1%)	18(6.3%)	24(5.6%)
Lifetime history of STD	Yes	14(9.6%)	17(5.6%)	31(7.2%)
	No	132(90.4%)	267(94.4%)	399(92.8%)
A lifetime number of sexual partners	1	47(32.2%)	132(46.5%)	179(41.6%)
	> 1	99(67.8%)	152(53.5%)	251(58.4%)
Do you use contraceptive	Yes	131(89.7%)	216(76%)	347(80.7%)
	No	15(10.3%)	68(24%)	83(19.3%)
Duration of contraceptive use	No	15(10.27%)	69(24.3%)	184(19.6%)
	< 5	119(81.5%)	146(51.4%)	265(61.6%)
	> = 5	12(8.2%)	69(24.3%)	81(18.8%)
Do you have cervical cancer symptoms	Yes	34(23.3%)	25(8.8%)	59(13.7%)
	No	112(76.3%)	259(91.2%)	371(86.3%)

### Knowledge and attitude of study participants about cervical cancer screening

Knowledge and attitude on cervical cancer screening were assessed by summing up response values from the knowledge and attitude questions. Knowledge about cervical cancer screening was analyzed as a continuous variable with a mean knowledge score of 9.5. 112(75%) of the case and 70(24.6%) of the controls had good knowledge about cervical cancer screening. Out of the total respondents, 138(94.4%) of cases and 241(84.8%) of controls heard about cervical cancer screening. 99(67.8%) of cases and 79(27.8%) of controls know VIA as one of the cervical cancer screening methods while 96(65.7%) of cases and 76(26.7%) of controls know pap smear as one of the cervical cancer screening method (Table 3).

**Table 3:** Knowledge and attitude of women attending health facilities of Dessie town, Northeast Ethiopia 2020

Variable	Characteristics	Cervical cancer screening		Total
		Screened(cases)	Not screened(controls)	
Knowledge	Good Knowledge	34(23.2%)	214(75.3%)	248(57.6%)
	Poor knowledge	112(76.8%)	70(24.7%)	182(42.3%)
Attitude	Positive attitude	31(21.2%)	221(77.8%)	252(58.6%)
	Negative attitude	115(78.8%)	63(22.2%)	178(41.4%)
Know women screened for CCS	Yes	119(81.5%)	196(69%)	315(73.2%)
	No	27(18.5%)	88(31%)	115(26.8%)
Recommended by a physician for CCS	Yes	73(50%)	24(8.5%)	97(22.6%)
	No	73(50%)	260(91.5%)	333(77.4%)
Is there a barrier for CCS	Yes	27(18.5%)	53(18.6%)	80(18.6%)
	No	119(81.5%)	231(81.4%)	350(81.4%)

Attitude towards cervical cancer screening was analyzed as a continuous variable with a mean attitude score of 4.5. Out of the total respondents, 115(78.7%) of cases and 63(22.2%) of controls had positive attitude scores regarding cervical cancer screening.141 (96.5%) of cases and 241(84.8%) of controls believed that cervical cancer is a killer if not detected early. 91(62.3%) of cases and 58(20.4%) of controls believe that cervical cancer is not communicable. 85(58.2%) of cases and 67(23.5%) of controls believe women should be screened for cervical cancer. 92(63%) of cases and 66(23.2%) of controls believe that cervical cancer screening can find changes in the cervix before they become cancerous.

### Determinant of cervical cancer screening

This study tries to assess the determinants of cervical cancer screening. A comparison of variables tested in the bivariate logistic regression analysis was entered into multivariable logistic regression analysis, those with a *p*-value less than or equal to 0.05, and adjusted (Table 4). Controlling for the effect of other confounding factors age group of 35 and more, current occupation with the private employee, having symptoms of vaginal bleeding or pelvic pain or post-coital bleeding or vaginal discharge, being recommended by a physician for screening, and having a positive attitude towards cervical cancer screening were found to be significantly associated with utilization of cervical cancer screening.

**Table 4:** Determinant of cervical cancer screening among women attending health facilities of Dessie town, Northeast Ethiopia 2020

Variable	Characteristics	Cervical cancer screening		COR (95%CI)	AOR (95%CI)
		**Screened(cases)**	**Not screened (controls)**		
Age in years	< 35	22(15.1)	207(72.9)	1	1
	> = 35	124(84.9)	77(27.1)	15.15(8.97–25.57)	11.52(6.09–21.77)*
Residence	Urban	101(69.2%)	139(48.9%)	2.34(1.53–3.56)	1.79(0.84–3.8)
	Rural	45(30.8%)	145(51.1%)	1	1
Educational status	No formal education	16(11%)	52(18.3%)	1	1
	Primary	31(21.2%)	74(26.1%)	1.36(0.67–2.74)	1.22(0.45–3.33)
	Secondary	64(43.8%)	127(44.7%)	1.63(0.86–3.09)	2.35(0.8–6.85)
	College and above	35(24%)	31(10.9%)	3.67(1.75–7.69)	3.28(0.76–14.05)
Occupation	Housewife	47(32.2%)	201(70.8%)	1	1
	Gov.t employee	20(13.7%)	23(8.2%)	3.71(1.88–7.32)	1.4(0.52–3.71)
	Private employee	79(54.1%)	60(21%)	5.63(3.54–8.93)	3.08(1.37–6.95)*
Average monthly income	< 1000	8(5.5%)	95(33.4%)	1	1
	1000–1999	29(19.8%)	73(27.7%)	4.71(2.03–10.92)	2.17(0.7–6.7)
	≥ 2000	109(74.7%)	116(40.9%)	11.58(5.18–24.04)	2.52(0.88–7.25)
number of sex partners	1	47(32.2%)	132(46.5%)	1	1
	> 1	99(67.8%)	152(53.5%)	1.82(1.2–2.77)	0.74(0.36–1.5)
Contraceptive use	Yes	131(89.7%)	216(76%)	2.74(1.5–5)	0.00
	No	15(10.3%)	68(24%)	1	1
contraceptive use in years	0	15(10.27%)	69(24.3%)	1	1
	1–4	119(81.5%)	146(51.4%)	3.74(2.04–6.89)	0.68(0.24–1.9)
	> = 5	12(8.2%)	69(24.3%)	0.8(0.34–1.83)	0.9(0.32–2.49)
Do you have cervical cancer symptoms	Yes	34(23.3%)	25(8.8%)	3.14(1.79–5.51)	3.08(1.37–6.5)*
	No	112(76.3%)	259(91.2%)	1	1
Know women screened for CCS	Yes	119(81.5%)	196(69%)	1.97(1.21–3.22)	0.76(0.36–1.6)
	No	27(18.5%)	88(31%)	1	
Recommended by a physician for CCS	Yes	73(50%)	24(8.5%)	10.83(6.38–18.39)	3.07(1.45–6.49)*
	No	73(50%)	260(91.5%)	1	1
Knowledge	Good Knowledge	112(76.8%)	70(24.7%)	10.07(6.29–16.1)	1.27(0.42–3.78)
	Poor knowledge	34(23.2%)	214(75.3%)	1	1
Attitude	Positive attitude	115(78.8%)	63(22.2%)	13.01(8–21.14)	5.33(2.8–10.59)*
	Negative attitude	31(21.2%)	221(77.8%)	1	1

^*^Significant at *P*-value < 0.05

Women of age 35 years and more were 11 times more likely to be screened for cervical cancer than those who were in the age group of less than 35 years [AOR] = 11.24 95%CI:5.49- 23.43). Current occupations of private employees were 4.67 times more likely to utilize cervical cancer screening than those of housewives (AOR = 4.67 95%CI: 2.41–9.03).

Women having symptoms of vaginal bleeding or pelvic pain or post-coital bleeding or vaginal discharge were3.08 times more likely to utilize the cervical cancer screening than those who did have not those symptoms (AOR = 3.08 95%CI: 1.37–6.95). Those who were recommended by health professionals for CCS were 3.07 times more likely to utilize cervical cancer screening than those who were recommended (AOR = 3.07 95%CI: 1.45, 6.49). Having a positive attitude toward CCS was 5.3 times more likely to utilize cervical cancer screening than those who have a negative attitude toward CCS(AOR = 5.3(2.8–10.59) (Table 4).

## Discussion

This study showed that maternal age was one of the significant determinants of cervical cancer screening utilization. Women in the age group of 35 and more were 11 times more likely to be screened as compared to women in the age group of less than 35.

One possible explanation is that symptoms of cervical cancer usually appear in women over the age of 30. Another explanation could be that Ethiopia's health policy encourages the screening of women over the age of 30. It could also be that as they get older, they may have multiple births, so they go to medical facilities for prenatal care, postnatal care, and delivery, while also receiving information about cervical cancer.

Schools and higher education institutions should widely utilize comprehensive sexuality and life skills education. Additionally, out-of-school platforms such as youth centers, peer clubs, associations, and the media should provide spaces for education on cervical cancer screening.

Women with current occupations as private employees were 4.67 times more likely to be screened as compared with housewives. This is consistent with a study conducted in Bishoftu [18], Addis Ababa [9], and the Tigray region [17] which shows employed women were more likely to have the intention to screen.

This is likely because employed women have their source of income so that they can consider their health issues as a priority. In addition, it could be explained as employed women have more exposure to information and have different sources from which they can gather information, unlike housewives.

Women having symptoms of vaginal bleeding or pelvic pain or post-coital bleeding or vaginal discharge were 3 times more likely to utilize the cervical cancer screening than those who do have not those symptoms. This was not supported by other research. It could be supported by the fact that women that know those symptoms as cervical cancer symptoms tend to be screened if they have experienced it because they feel they could be exposed to cervical cancer.

In the study, women who were referred for cervical cancer screening by a healthcare professional were three times more likely to be screened for cervical cancer than women who were not referred by a healthcare professional. This may be because women who regularly visit health facilities have excellent opportunities to discuss a variety of health issues with health professionals, including the reproductive health component, where clients will receive information about cervical cancer and cervical cancer screening that initiates them to utilize the service. This is similar to a study done in Uganda [19], Debre Markos [20].

The finding that women who were referred for screening by a healthcare professional were more likely to be screened for the disease suggested that most women were screened only at the direction of a healthcare professional. This is both a challenge and an opportunity. One challenge is that cervical cancer is often diagnosed at an advanced stage when most women seek services too late. The opportunity is that health workers can be an effective intervention to increase women's use of screening services. The women in this study reported that health workers were an important source of cervical cancer screening information.

When cervical cancer screening is available, the information received from healthcare providers can significantly impact its acceptance. Therefore, increasing awareness of this group of providers is critical to the success of any public health program.

Attitudes of participants were a major factor influencing cervical cancer screening. Participants with positive attitudes were screened 5.3 times more often than those with negative attitudes. The common reason for this might be that those participants who had a positive attitude toward cervical cancer might have believed that cervical cancer screening would protect them from developing cervical cancer. This finding is consistent with a study conducted in Ilorin, Nigeria [21], Tigray region, Ethiopia [17], and Debre Markos [20].

## Conclusion

In this study, women in the age group of 35 and above, current occupation with a private employee, a Positive attitude toward cervical cancer screening, is recommended by a physician for screening, and having symptoms of cervical cancer were significantly associated with utilization of cervical cancer screening utilization. Health professionals should incorporate cervical cancer and cervical cancer screening utilization in their day-to-day health education program. Specifically, health professionals in Maternity service delivery should make one-to-one health information delivery on cervical cancer and cervical cancer screening and behavioral change communication to create a positive attitude toward cervical cancer screening.

## Supplementary Information

**Additional file 1.**

## Data Availability

All the necessary data are included in the manuscript. An English version data collection tool and detailed operational definitions of the outcome variable are accessible at a reasonable request from the corresponding author.

## Abbreviations

CC: Cervical Cancer
CCS: Cervical Cancer Screening
COC: Combined Oral Contraceptive
ETB: Ethiopian Birr
GH: General Hospital
HIV: Human Immune-deficiency Virus
HPV: Human Papilloma Virus,
IRC: Institutional Review Committee
IUD: Intra Uterine Device
STD: Sexually Transmitted Diseases
SWZ: South Wollo Zone
VIA: Visual Inspection with Acetic acid
WHO: World Health Organization
