# Supplementary material for: Determinants of cervical cancer screening utilization among women attending health facilities of Dessie town, Northeast Ethiopia
Source: BMC Cancer. 2022 Dec 20;22:1330. doi: 10.1186/s12885-022-10447-0 (PMC9764547; doi:10.1186/s12885-022-10447-0)
Supplement: Supplementary file 1 — Additional file 1. [file 12885_2022_10447_MOESM1_ESM.docx]

**Questionnaire (English)**

**Questionnaire to assess the determinants of cervical cancer screening among women attending health facilities of Dessie town, south wollo zone Ethiopia 2012 E.C.**

Do you come here for cervical cancer screening?

1. Yes
2. No

| **Part I: Socio-Demographic Characteristics of respondents** | | |
| --- | --- | --- |
| S.N | Questions | Codes and Answers |
| 101 | Age of respondents | …………(years) |
| 102 | Religion | 1. Orthodox  2. Muslim  3. Protestant  4. Others ____ |
| 103 | Residence | 1. Rural  2. Urban |
| 104 | Ethnicity | 1. Amhara  2. Others______ |
| 105 | Marital status | 1. marred  2. single  3. divorced  4. widowed |
| 106 | Educational status | 1.no formal education  3. primary (1-8)  3. secondary (9-12)  4. College/University and above |
| 107 | Occupation | 1. House Wife  2. Gov,t employer  3. Private Employer |
| 108 | Average monthly income | ------------------eth birr |

**Section 2: Behavioral, gynecologic, and obstetric history of respondents to assess the determinants of cervical cancer screening among women attending health facilities of Dessie town, South Wollo Zone; Ethiopia 2012 E.C**

| 201 | Age at first sexual intercourse | ------------------ |
| --- | --- | --- |
| 202 | Have you given birth | 1. Yes 2. No |
| 203 | If yes how many | ----------------- |
| 204 | Have you ever tested for HIV | 1. Yes 2. No |
| 205 | If yes, what was the result | 1. positive  2. negative |
| 206 | Lifetime history of STD | 1. Yes 2. No |
| 207 | A life time number of sexual partners | ------------------- |
| 208 | Do you use contraceptive | 1. Yes  2. No |
| 209 | If your answer is yes for Q208, for how many years have you used | ------------------ |
| 210 | History of smoking | 1. Yes 2. No |
| 211 | If your answer is yes for Q211, for how many years | -------------------- |
| 212 | Do you have one of the following symptoms: vaginal bleeding or vaginal discharge or pelvic pain or postcoital bleeding | 1. Yes 2. No |

**Section 3: Knowledge-related questions about cervical cancer screening; to assess the determinants of cervical cancer screening among women attending health facilities of Dessie town, South Wollo Zone; Ethiopia 2012 E.C.**

| 301 | Have you ever heard about cervical cancer? | 1. Yes 2. No |
| --- | --- | --- |
| 302 | Do you know vaginal bleeding is a symptom of cervical cancer? | 1. Yes 2. No |
| 303 | Do you know pelvic or back pain is a symptom of cervical cancer? | 1. Yes 2. No |
| 304 | Do you know foul-smelling vaginal discharge is a symptom of cervical cancer? | 1. Yes 2. No |
| 305 | Do you know Post coital bleeding is  Symptoms of cervical cancer? | 1. Yes 2. No |
| 306 | Do you know Early onset of sexual intercourse is the risk factor for cervical cancer? | 1. Yes 2. No |
| 307 | Do you know Having multiple sexual partners is the risk factor for cervical cancer? | 1. Yes 2. No |
| 308 | Do you know the Family history of cervical cancer is the risk factor for cervical cancer? | 1. Yes 2. No |
| 309 | Do you know Cigarette smoking is a risk factor for cervical cancer? | 1. Yes 2. No |
| 310 | Do you know Avoiding multiple sexual partners is one way of preventing cervical cancer? | 1. Yes 2. No |
| 311 | Do you know Avoiding early-onset sexual intercourse is one way of preventing cervical cancer? | 1. Yes 2. No |
| 312 | Do you know being a non smoker is one way of preventing cervical cancer? | 1. Yes 2. No |
| 313 | Do you know vaccination is one way of preventing cervical cancer? | 1. Yes 2. No |
| 314 | Do you know screening is one way of preventing cervical cancer? | 1. Yes 2. No |
| 315 | Is cervical cancer curable? | 1. Yes 2. No |
| 316 | Do you know Seeking treatment at an early stage makes cervical cancer curable once diagnosed? | 1. Yes 2. No |
| 317 | Have you ever heard about cervical cancer screening | 1. Yes  2.No |
| 318 | Do you know VIA is one of the cervical cancer screening methods? | 1. Yes 2. No |
| 319 | Do you know Pap smear is one of the cervical cancer screening methods? | 1. Yes 2. No |
| 320 | Do you know the HPV test is one of the cervical cancer screening methods? | 1. Yes 2. No |
| 321 | Do you know women should be screened for cervical cancer once every 3 years? | 1. Yes 2. No |
| 322 | Do you know a woman should start screening for cervical cancer As soon as she becomes sexually active? | 1. Yes 2. No |
| 323 | Do you know the aim of cervical cancer screening is for detecting cervical cancer early? | 1. Yes 2. No |
| 324 | Do you know the aim of cervical cancer screening is for preventing cervical cancer? | 1. Yes 2. No |

**Part 4: Attitude assessment tool on cervical cancer screenings to assess the determinants of cervical cancer screening among women attending health facilities of Dessie city, South Wollo Zone; Ethiopia 2012 E.C.**

| 401 | Cervical cancer is a killer disease? | 1. Strongly agree 2. Agree 3. Undecided 4. Disagree 5. Strongly disagree |
| --- | --- | --- |
| 402 | Any reproductive-age women including you, are at risk of developing cervical cancer? | 1. Strongly agree 2. Agree 3. Undecided 4. Disagree 5. Strongly disagree |
| 403 | Screening is important in preventing cervical cancer. | 1. Very important 2. Important 3. Moderately important 4. Slightly important 5. Unimportant |
| 404 | All eligible women should be screened for cervical cancer. | 1. Strongly agree 2. Agree 3. Undecided 4. Disagree 5. Strongly disagree |
| 405 | You can have cervical cancer but no symptoms | 1. Always true 2. Usually true 3. Occasionally true 4. Usually not true 5. Never true |
| 406 | Cervical cancer is not communicable | 1. strongly agree  2. agree  3. disagree  4. strongly disagree |
| 407 | Screening cause no harm to the client | 1. strongly agree  2. agree  3. disagree  4. strongly disagree |
| 408 | Cervical cancer screening can find changes in the cervix before they become cancer | 1. strongly agree  2. agree  3. disagree  4. strongly disagree |
| 409 | If Cervical Changes Are Found Early from Cervical Cancer Screening, They Are Easily Curable. | 1. strongly agree  2. agree  3. disagree  4. strongly disagree |
| 410 | Cervical cancer develops slowly and is Preventable. | 1. strongly agree  2. agree  3. disagree  4. strongly disagree |

**Part 5; Factor related to accessibility of cervical cancer screening information and service**

| 501 | Have you ever known women who screened for cervical cancer? | 1. Yes  2 . No |
| --- | --- | --- |
| 502 | Do you have a family history of cervical cancer? | 1 Yes  2. No |
| 503 | Has your physician ever recommended cervical cancer screening? | 1. Yes  2. No |
| 504 | Even if you wanted to get a screening, is there a barrier | 1. Yes  2. No |
| 505 | If yes mention | 1. Unavailability of H. facilities  2. Unavailability of skilled professionals in HF  3. I cannot pay for services  4. The procedure is pain full  5. Fear of viginal exam  6. Lack of partner approval  7. Attitude of health provider  8. I am not at risk |
